# Phenotypic variation and epigenetic insight into tissue culture berry crops

**DOI:** 10.3389/fpls.2022.1042726

**Published:** 2022-12-19

**Authors:** Samir C. Debnath, Amrita Ghosh

**Affiliations:** ^1^ St. John’s Research and Development Centre, Agriculture and Agri-Food Canada, St. John’s, NL, Canada; ^2^ Department of Biology, Memorial University of Newfoundland, St. John’s, NL, Canada

**Keywords:** antioxidants, clonal fidelity, DNA methylation, *in vitro* culture, vegetative growth, somaclonal variation

## Abstract

Berry crops, a nutrient powerhouse for antioxidant properties, have long been enjoyed as a health-promoting delicious food. Significant progress has been achieved for the propagation of berry crops using tissue culture techniques. Although bioreactor micropropagation has been developed as a cost-effective propagation technology for berry crops, genetic stability can be a problem for commercial micropropagation that can be monitored at morphological, biochemical, and molecular levels. Somaclonal variations, both genetic and epigenetic, in tissue culture regenerants are influenced by different factors, such as donor genotype, explant type and origin, chimeral tissues, culture media type, concentration and combination of plant growth regulators, and culture conditions and period. Tissue culture regenerants in berry crops show increased vegetative growth, rhizome production, and berry yield, containing higher antioxidant activity in fruits and leaves that might be due to epigenetic variation. The present review provides an in-depth study on various aspects of phenotypic variation in micropropagated berry plants and the epigenetic effects on these variations along with the role of DNA methylation, to fill the existing gap in literature.

## Introduction

Berry crops, also called small fruits, are composed of plants in a number of genera including *Actinidia* (hardy kiwi), *Amelanchier* (Juneberry/Saskatoon, serviceberry), *Aronia* (*Aronia melanocarpa*), *Fragaria* (strawberry), *Hippophae* (sea buckthorn), *Lonicera* (edible honeysuckle/Haskap), *Prunus* (chokecherry), *Ribes* (currants, gooseberries), *Rubus* (blackberries, raspberries and their hybrids), *Sambucus* (elderberries), *Schisandra* (schisandra), *Shepherdia* (silver buffalo berry), *Vaccinium* (blueberries, cranberries, lingonberries, bilberries, and huckleberries), *Viburnum* (highbush cranberry), and *Vitis* (grapes) (Debnath et al., 2012; Debnath, 2016; Debnath, 2018). Although grapes are the most important berry crop worldwide, the production of strawberries, blueberries, raspberries, and cranberries is also a profitable agricultural enterprise (Debnath, 2016). These produce small- to moderate-sized edible fruits on perennial vines, herbs, or shrubs, and can be consumed in fresh, juice, dried, or processed form. The fruits and leaves are a good source of health-promoting bioactive compounds (Vyas et al., 2015; Abeywickrama et al., 2016; Debnath and An, 2019; Bhatt and Debnath, 2021) and are believed to fight against cardiovascular and degenerative diseases (Isaak et al., 2017; Debnath, 2018; Debnath and Arigundam, 2020; Debnath and Goyali, 2020; Hewage et al., 2020). Berry crops are genetically heterozygous and are generally propagated vegetatively to maintain genetic integrity and fruit quality, and for achieving quick fruit-bearing condition (Debnath, 2018). Micropropagation, also called *in vitro* propagation, is a multi-billion-dollar industry across the world for the propagation of various crop plants. It has been long used in berry crops for their quick and mass propagation in commercial cultivation. Despite the tremendous advantage of this propagation system in berry crop plants, clonal fidelity or trueness-to-type is of significant importance for its commercial application. *In vitro*-derived variations can be heritable (genetic) or epigenetic, that arise without alteration of DNA sequences but due to gene activation or silencing. Epigenetic variations can be unstable and may disappear following transfer from culture media or after a few clonal or sexual generations (Kaeppler et al., 2000), although heritable epigenetic changes are also available that are valuable for enhanced crop production and improvement (Kakoulidou et al., 2021). However, propagation strategies have been developed to minimize variations in micropropagated berry plants (Debnath and Goyali, 2020). Although a number of DNA-based molecular markers have been used to monitor the clonal fidelity in berry crops including raspberry (Debnath, 2014), lingonberry (Arigundam et al., 2020), blueberry (Goyali et al., 2015; Debnath, 2017a), and strawberry (Debnath, 2009a), tissue culture-induced epigenetic variation such as DNA methylation has been studied so far only in blueberry (Ghosh et al., 2017; Goyali et al., 2018; Ghosh et al., 2021a), strawberry (Cao et al., 2021), and lingonberry (Sikdar et al., 2022). The present review deals with in-depth variations during *in vitro* culture of berry crops along with their analysis at morphological, biochemical, and molecular levels.

## *In vitro* propagation

The theoretical concept of *in vitro* culture was explained by Gottlieb Haberlandt in his lecture at the German Academy of Sciences based on his experiments on single-cell culture (Haberlandt, 1902). Haberlandt experimented with leaf and other actively differentiating cells that failed to give rise to artificial embryos. He further added that cultivating vegetative cells under optimal hormonal influence can be established as a new approach for plant propagation. Although he did not succeed in his experiments, his practice of regeneration of artificial embryos from vegetative cells supported the concept of totipotency (Thorpe, 2007). Haberlandt (1902) was successful at tuning the survival of *in vitro*-grown tissue, while cell division under *in vitro* conditions was first observed by Hannig (1904) and regeneration from callus tissue was first documented by Simon (1908). However, Gautheret (1934) demonstrated the first true plant tissue cultures using cambial tissue of *Acer pseudoplatanus*.

Regeneration of plants *via* tissue culture is dependent on two primary concepts, which are totipotency and developmental plasticity. According to Skoog and Miller (1957), totipotency can be explained as the ability of a plant cell to differentiate, proliferate, and eventually grow into a plantlet under optimal culture conditions and under hormonal influence. In general, cells from young tissues and meristems are totipotent in nature; however, differentiated cells also occasionally exhibit totipotency (Debnath, 2007a). On the other hand, plasticity is the capability of the plant tissues to adjust their metabolism, growth pattern, and development in order to survive under various environmental conditions. Plant tissue culture, which is also known as *in vitro* cell culture, is an important area of basic and applied science studies. Due to the availability of several *in vitro* propagation techniques, since approximately mid-1960s to 1980s, these techniques have gained a huge popularity to solve various biological, agricultural, horticultural, and forestry problems (Thorpe, 2007).

During the *in vitro* culture process, an individual cell, tissue, or organ of a plant can be used as an explant. Explants are then cultured in an artificial medium containing macro- and micronutrients, a carbohydrate source, vitamins, plant growth regulators (PGRs), and a chelating agent depending on media type. Under the optimum hormonal stimuli and appropriate environment, explants develop into an identical copy of the source plant under aseptic conditions, which is called a clone (Altman and Loberant, 2010). Clones can be regenerated during *in vitro* propagation *via* organogenesis by forming either shoot or root meristems and/or somatic embryogenesis (SE) where shoot and root meristems form simultaneously (Steward et al., 1970). Organogenesis follows the pathway of shoot proliferation from a pre-existing bud followed by adventitious shoot regeneration (Steward et al., 1970). In a tissue culture system, plants produce hundreds of identical copies within a short period. The commercial use of micropropagation techniques includes the maintenance of pathogen-free germplasm, production of nuclear stock, and yearlong production of clones of hybrid and parental lines (Debnath, 2014). In recent years, for the large-scale commercial micropropagation of elite varieties, industries have implemented micropropagation techniques. Consequently, many tissue culture laboratories were set up around the globe especially in developing countries due to cheaper labor costs (Kumar and Reddy, 2011). *In vitro* propagation of blueberry was first reported in the early 1970s using rhizome explants without the help of any PGR (Barker and Collins, 1963) in White’s medium (White, 1943). However, the first report on the use of micropropagation techniques for commercial purposes was in the 1970s by Boxus (1974) in strawberry. Some other major discoveries were chemical and hormonal regulation of plant regeneration (Skoog and Miller, 1957), the application of *in vitro* propagation techniques in basic and applied science (Komamine et al., 1992), regeneration of virus-free plantlets, haploid culture (Nitsch and Nitsch, 1969), plantlet formation from protoplast culture (Cocking, 1960), secondary metabolite production (Kaul and Staba, 1965), and cell culture in a liquid medium in a bioreactor (Noguchi et al., 1977). Skoog and Miller (1957) hypothesized the effect of auxin–cytokinin on plant morphogenesis. The authors concluded from their experiment on tobacco pith culture that the auxin–cytokinin ratio is the deciding element for shoot and root formation. Skoog and Miller (1957) hypothesized that the culture medium supplemented with a higher concentration of cytokinin gives rise to shoot, and an increased concentration of auxin induces root formation, whereas a balanced ratio of auxin and cytokinin leads to the formation of a callus.

Micropropagation in berry crops is being used for year-round propagation of virus-free (indexed) clones, which can be used as a first step in a nuclear stock crop production system (Debnath, 2018). Softwood or rhizome cuttings of desirable clones can be used to establish new blueberry stands as they are comparatively easy to root; however, their flowering ability makes the establishment process very slow for plantings (Debnath, 2014). With the intervention of micropropagation techniques, this problem can be largely avoided (Smagula and Lyrene, 1984). Many of the small fruit crops have been traditionally produced *via* plant tissue culture techniques to attain rapid fruit-bearing condition and to maintain genetic fidelity (Debnath, 2018). Especially in the case of lowbush blueberry, micropropagated plants develop similar spreading behaviors as seedlings. In addition to that, they exhibit consistent productivity behaviors similar to the rooted cuttings (Frett and Smagula, 1983). Furthermore, *in vitro* propagation methods match the traditional way of introducing new desirable characters into the progeny and multiplying them in a short period of time (Meiners et al., 2007). Propagation *in vitro* has been reviewed in berry crops (Debnath, 2018; Debnath and Keske, 2018; Debnath and Arigundam, 2020; Debnath and Goyali, 2020).

There are three ways of propagation *in vitro*: axillary shoot proliferation, adventitious shoot regeneration, and SE. Shoot proliferation from axillary buds is one of the most common pathways of organogenesis to attain true-to-type clones from the donor plants due to low chances of genetic alterations (Debnath, 2018). It does not involve cell differentiation; rather, it follows the natural pathway of plantlet formation from pre-existing meristems (Novikova and Zaytseva, 2018). Meristem tissues are comparatively less prone to mutations or genetic alteration than unorganized tissues such as callus; however, the chance of changes occurring during *in vitro* culture still persists (Baránek et al., 2015). Berry crop micropropagation *via* axillary shoot proliferation has been reported by various authors both on semisolid media (Chauhan et al., 2019; de Oliveira Prudente et al., 2019; Kopper et al., 2020; Kryukov et al., 2022) and in liquid media using bioreactor systems (Arencibia et al., 2013a; Arencibia et al., 2013b; Debnath, 2017a; Arigundam et al., 2020; Ghosh et al., 2021a; Kryukov et al., 2022).

**Figure 1:**
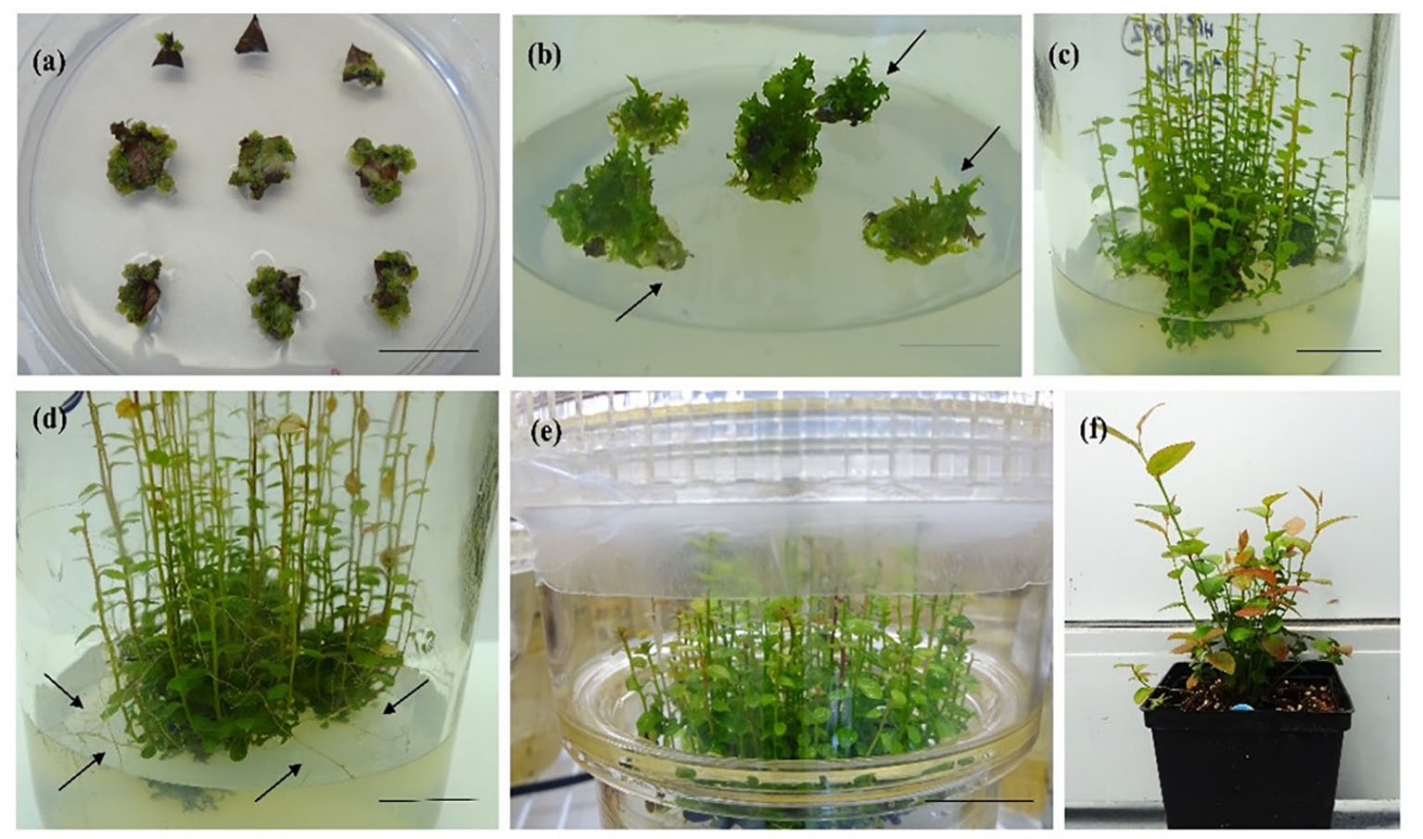
*In vitro* organogenesis in half-high blueberry cv. “Patriot”. **(A)** Callus formation after 4 weeks of culture on a semisolid medium (SSM) with 2.3 µM ZEA (bar = 2 cm), **(B)** bud initiation from callus (arrows) after 8 weeks of culture on SSM with 9.2 µM zeatin (bar = 2 cm), **(C)** shoot regeneration after 12 weeks of culture on SSM with 9.2 µM zeatin (bar = 2 cm), **(D)** shoot elongation and root formation (arrows) after 16 weeks of culture on SSM with 9.2 µM zeatin (bar = 2 cm), **(E)** shoot elongation after 16 weeks of culture in a temporary immersion bioreactor (TIB) containing a liquid medium with 9.2 µM zeatin (bar = 3 cm), and **(F)** 1-year-old hardened-off plants in a greenhouse (bar = 8 cm).

**Figure 2:**
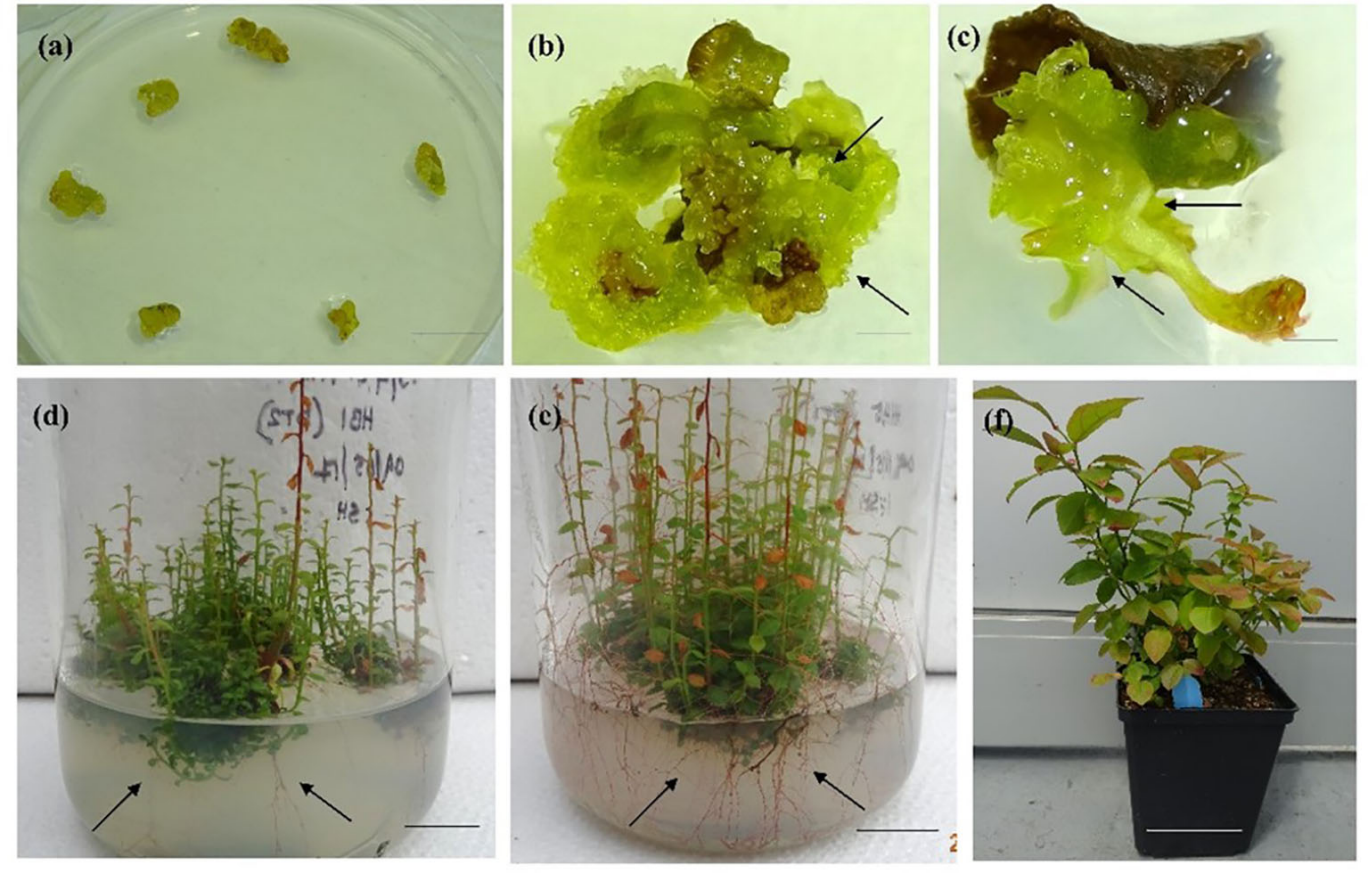
*In vitro* propagation *via* somatic embryogenesis in half-high blueberry cv. “Northblue”. **(A)** Young leaf cultured on a medium with 9 µM TDZ, **(B)** globular embryo development (arrows) after 4 weeks of culture in a 9 µM TDZ-supplemented medium, **(C)** germination of somatic embryos and shoot and root apex development (arrow) after 10 weeks of culture with 9 µM TDZ (scale bar = 0.5 cm), **(D)** root elongation (arrows) in a nutrient medium containing 2.3 µM TDZ, after 4 weeks of culture in a glass jar (scale bar = 2 cm), **(E)** development of rooting system (arrows) in a nutrient medium with 2.3 µM TDZ after 6 weeks of culture in a glass jar (scale bar = 2 cm), and **(F)** 1-year-old hardened-off plants in a greenhouse (scale bar = 5 cm).

*De novo* adventitious shoot regeneration can occur two ways: directly without any involvement of callus, or indirectly *via* callus formation. However, direct shoot regeneration is the more preferable technique for mass propagation as the chances of somaclonal variance are scarce (Debnath, 2018; Novikova and Zaytseva, 2018). During the indirect regeneration process, shoots originate from the surface of the callus tissue and thus have a better chance to be genetically transformed (Arencibia et al, 2013b). According to Ammirato (1985), adventitious shoot regeneration can initiate from unipolar organs either through shoot or with root meristem (organogenesis; **Figure 1**, Ghosh et al, 2021a) or from bipolar organs simultaneously containing shoot and root meristem (SE; **Figure 2**, Ghosh et al., 2018). The adventitious shoot regeneration technique has been successfully employed for mass propagation of berry crops using semisolid (Qiu et al, 2018; Schuchovski et al., 2020) and liquid media in bioreactors (Debnath, 2014; Arigundam et al., 2020). While working with blackberry (*Rubus fruticosus*), blueberry (*V. corymbosum*), and kiwiberry (*Actinidia argute*), Hunková et al. (2022) regenerated shoots from petioles and internodal segments; Verma et al. (2022) reported plant regeneration through adventitious shoot formation from hypocotyl and leaf explants of *Lycium barbarum* (Goji), on a semisolid medium.

SE is explained as the process of formation of embryos from differentiating somatic cells. Somatic embryos and zygotic embryos are remarkably similar both temporally and morphologically, although they develop independent to the physical constraints of the maternal tissues (Zimmerman, 1993). Somatic embryos are bipolar and contain radicals, hypocotyl, cotyledons, and the usual embryonic organs (von Arnold et al., 2002). Similar to zygotic embryos, somatic embryos also develop globular-shaped, heart-shaped, and torpedo-shaped embryos followed by formation of shoot and root meristem simultaneously (Zimmerman, 1993). Like organogenesis, SE can be direct from an explant or indirect, involving callus formation (Williams and Maheswaran 1986). Microspores, ovules, embryos (zygotic and somatic), and seedlings are a few examples of explants that are commonly used in direct embryogenesis (von Arnold et al., 2002). The morphological differences between direct and indirect SE are still not clear. However, according to an older hypothesis, direct SE starts from pre-destined embryonic cells while indirect SE is induced from undetermined unorganized callus tissues. However, calluses developed during the indirect SE process can be either embryogenic or non-embryogenic (von Arnold et al., 2002).

The initial description of SE was reported on carrot callus culture (Steward et al., 1958), and since then, carrot has been used as the principal model system to study the early regulatory and morphogenic processes occurring during SE (Zimmerman, 1993). SE is a unique developmental pathway that is well recognized as a plantlet regeneration pathway from cell culture systems (Zimmerman, 1993). There are few available detailed reports on SE in blueberries (Ghosh et al., 2017; Ghosh et al., 2018). However, studies are available on SE in grapes (Nakajima and Matsuta, 2003; Dhekney et al., 2016; Forleo et al., 2021), strawberry (Biswas et al., 2009), *Arbutus unedo* (strawberry tree; Martins et al, 2022) and *L. barbarum* (Verma et al., 2022) on semisolid media. Reports on SE in berry crops are scarce in a bioreactor system.

## Liquid media and bioreactors for propagating berry crops

Micropropagation of berry crops using liquid media in a bioreactor system is one of the most developed techniques of mass propagation. Over time, automated bioreactors have become an important tool for the success of large-scale commercial production of tissue culture plants (Debnath, 2014). Haberlandt (1902) first used sucrose supplemented in Knop’s liquid medium for propagating individual bract cells of Lamium purpureum (Preil, 2005). Kohlenbach (1959) used mesophyll cells of Macleaya as an explant to develop cell-forming organs, somatic embryos, and cell clusters 60 years after Haberlandt’s experiment. Liquid media have been used in stationary and temporary immersion bioreactors (TIBs) to induce organogenesis and SE (Ziv, 2005). Paek et al. (2005) described bioreactors as a sterilized, independent unit that works on the principal of inflow and outflow of the liquid medium. Automated bioreactors are capable of easily managing the microenvironment conditions such as aeration, agitation, and dissolved oxygen level during the intensive culture process (Paek et al., 2005). The environment of the culture room is also responsible for determining the microenvironment inside the bioreactor (Morini and Melai, 2003). Bioreactors also provide a better control of the gaseous exchange of plants, pH of the medium, and temperature (Levin and Tanny, 2004). Keeping the above-mentioned factors in mind, two types of bioreactors have been developed: (a) agitated and (b) non-agitated bioreactors (Debnath, 2011a).

Levin and Vasil (1989) introduced bioreactor systems for mass propagation of various horticultural crops: ever since, agitated and non-agitated bioreactors have been used successfully for various ornamental and vegetative crops such as oriental lily (Lian et al., 2003) and potato (Piao et al., 2003). Micropropagation of berry plants using liquid medium in bioreactors has been investigated in few berry crops (Debnath, 2007; Debnath, 2008; Debnath, 2009a; Debnath, 2009b; Debnath, 2011a; Debnath, 2011b; Arencibia et al., 2013a; Arencibia et al., 2013b; Debnath, 2014; Debnath, 2017a; Arencibia-Rodríguez et al., 2018; Arigundam et al., 2020; Kryukov et al., 2022). A two-step procedure for cloudberry (*Rubus chamaemorus* L.) micropropagation using a liquid medium in plastic airlift bioreactors was reported by Debnath (2007). Strawberry shoot culture was obtained in commercially available TIBs using leaves from five cultivars as explants in Murashige and Skoog (MS) medium supplemented with 9 µM thidiazuron (TDZ) and 2.5 µM indole-3-butyric acid (IBA) (Hanhineva et al., 2005). Debnath (2008) optimized the shoot regeneration and proliferation protocol in strawberry cv. “Bounty” using a liquid medium in a TIB system coupled with a semisolid medium (SSM) with 2–4 µM TDZ. Similarly, micropropagation of two lowbush blueberry genotypes and one cultivar was established using TIB in combination with a SSM and 1, 2, or 4 µM zeatin (ZEA) (Debnath, 2009b). Adventitious shoots of three lowbush blueberry genotypes “PB1,” “QB1,” and “QB2” were regenerated in a liquid medium supplemented with 1.2–2.3 µM thidiazuron (TDZ) in a bioreactor (Debnath, 2011b). Arencibia et al. (2013b) reported micropropagation of three raspberry cultivars “Meeker,” “Amity,” and “Heritage” using shoot tip cultures (~5 cm) in a liquid media in a TIB supplemented with various sucrose concentrations. *In vitro* multiplication of raspberry cultivars “Festival,” “Heritage,” and “Latham” has been reported by Debnath (2014) in TDZ-supplemented media. Adventitious shoot regeneration of wild lingonberry clones were obtained using stationary bioreactors and TIBs with liquid media supplemented with 9.1 µM TDZ and 1.8 µM ZEA (Arigundam et al., 2020). Organogenesis in half-high blueberry cv. “Patriot” is shown in **Figure 1** using a semisolid and a liquid medium in a TIB (Ghosh et al., 2021a). Clapa et al. (2021) micropropagated raspberry cultivars “Maravilla” and “Willamette” in TIB containing a liquid MS medium supplemented with 0.5 mg/L 6-benzyladenine (BA). Kryukov et al. (2022) used bioreactor micropropagation in strawberry cultivar “Murano” and grapevine cultivar “Traminer Pink” in combination with a semisolid medium. Shoots were proliferated in the semisolid medium followed by proliferation in a liquid medium containing 2 mg/L BA. The authors reported a 300-fold fresh mass increase after a 6-week cycle in TIB with a propagation increase rate of approximately 5 and 20 for grape and strawberry, respectively (Kryukov et al., 2022).

## The epigenome of plants

Plant studies have provided a significant contribution in epigenetic research. In 1929, Heitz observed the difference between euchromatin and heterochromatin based on several cytological analyses (Heitz, 1929). Carl von Linne in the 18th century described “peloric” mutants with changed floral symmetry that was due to silenced epialleles mimicking the DNA sequence of the expressed alleles, as described by Cubas et al. (1999). A year after, Soppe et al. (2000) showed that epialleles can make changes in developmental switches. In *Arabidopsis thaliana*, the FWA gene is prominent evidence of this phenomenon. Paramutation in maize and tomato is another epigenetic phenomenon, which displays non-Mendelian epigenetic inheritance (Chandler et al, 2000). Later, paramutation has also been detected in mammals, flies, and plants (Cao et al., 2021). Understanding the paramutation phenomenon led several scientists who had been using plants as a model system to study epigenetic regulations. With the discovery of transposable elements in maize in the 1940s by McClintock and others, various connections were made between genetic and epigenetic regulations (Lisch, 2009). Mobilization of transposable elements has also been used as a technique to introduce epigenetic mutations in plant systems. Usually, engineered transgenes are used as silenced marker genes to screen mutations in epigenetic regulators and that is how the use of transgenic techniques in plant development proved to be advantageous in epigenetic research (Pikaard and Scheid, 2014).

## Molecular components of epigenetic regulation in plants

More than 130 protein-encoding genes have been found to control epigenetic regulators in plants to date (Pikaard and Scheid, 2014). During plant development, epigenetic regulations play an important role as they help the plant to maintain the stability and integrity of their gene expression profiles. There are several reported studies focusing on alteration of DNA methylation and histone modifications during the de-differentiation pathway in the tissue culture system (Ruffoni and Savona, 2013). Although there is an ever-developing flow of information on epigenetic modifiers, based on current knowledge, they are discussed in the following sections.

## DNA methylation

DNA methylation is one of the most studied epigenetic modifications. DNA methylation is a chromatin modification that does not alter genetic sequence but regulates gene expression by suppressing the transcription factor and DNA association (Smith and Meissner, 2013). DNA methylation can be present in various forms depending on the targeted nucleotide during the modification process (Ratel et al., 2006). It is a post-replicative mechanism and generates several methylated bases such as 5-methylated cytosine (5-mC), N6-methyladenine (6-mA), and N4-methylcytosine (4-mC) (Wion and Casadesús, 2006). 5-mC is available mostly in higher plants and mammals, unlike 6-mA and 4-mC, which are predominant in bacteria, protists, and lower eukaryotes (Wion and Casadesús, 2006). In the case of plants, cytosine methylation usually takes place within CG, CHG, or CHH motifs (H = A/T/C). However, in the case of mammals, promoters are usually present in CpG islands, although CpG islands are not easily detectable in plants. Cytosine methylation takes place randomly within the protein-coding regions of highly expressive genes, at their differentially regulated promoters in plants (Zilberman et al., 2007), and they also occur mainly in the repetitive parts of the genome such as in transposable elements and silenced rRNA gene repeats (Pikaard and Scheid, 2014).

Various DNA cytosine methylation enzymes such as methyltransferases (METS) and domain rearranged methyltransferases (DRMs) mediate the DNA methylation process (Rival et al., 2008). DNA methyltransferases facilitate the formation of newly methylated cytosines at the previously unmethylated cytosines or maintain a preexisting methylation pattern (Li and Zhang, 2014). In eukaryotes, there are three families of DNA methyltransferases that are conserved, and these three families are present in modern plants and are homologs of mammalian DNA methyltransferases such as Dnmt1, Dnmt2, and Dnmt3 (Li and Zhang, 2014). DNA methyltransferase 1 (MET 1), which is a plant equivalent of Dnmt1, carries out CG motif maintenance and contributes to *de novo* methylation of CG contexts (Zhang et al., 2018). On the other hand, plant homologs of Dnmt2 process the methylation activity of transfer RNA (Goll et al., 2006). DRMs, which are the plant equivalent of mammalian Dnmt3 groups, primarily carry out *de novo* cytosine methylation. Among others, DRM 2 catalyzes cytosine methylation of all CG, CGH, and CHH contexts and predominantly takes part in RNA-directed DNA methylation pathways (Pikaard and Scheid, 2014). In contrast to mammals, plants have an exclusive family of cytosine methyltransferase, which binds to the methylated histones at their chromodomains. Chromomethylase 1 (CMT 2) plays an important role in maintaining CHH methylation at the central part of the large transposons while CMT 3 is one such enzyme, which is mainly responsible for CHG maintenance methylation (Zemach et al., 2013). CMT 3 also takes part in the balancing act between repressive DNA methylation and histone modification by dimethylating histone 3 protein on lysine 9 (H3K9me2), thus maintaining the cross talk between both epigenetic states in plants (Law and Jacobsen, 2010).

## Histone modifications

Similar to any other organism, plants have several enzymes that post-translationally modify various histone proteins affecting gene regulation. In plants, these enzymes are generally encoded by large gene families (Berr et al., 2011). Histone modifications usually occur in two ways, namely, acetylation and methylation. Histone acetylation and histone methylation are reversible epigenetic marks, as enzymes like histone acetyltransferase (HAT) mediate “writer” activities, and histone deacetylase (HDAC) acts as an “eraser” of the acetylation phenomenon; similarly, histone lysine methyltransferases (HKMTs) also reverse the methylation phenomenon by methylating a specific lysine on histones, thus promoting or inhibiting the transcription process (Cheng, 2014). Plants have several HAT and HDAC gene families (Chen and Tian, 2007). The genes included in the HAT family can be classified into five subfamilies depending on their structure and substrate profiles (Earley et al, 2007). However, only two HDAC gene families have been identified to date: HDA1 and HDA6. While the function of HDAC1 is still unclear, HDAC6 is responsible for interacting with MET1, as well as the maintenance of CG and CHG methylation. HDAC6 is also responsible for transposon silencing, repression of rRNA genes, and nucleolar dominance (Pikaard and Scheid, 2014). Aufsatz et al., 2007 also found that HDAC6 actively takes part in seed maturation, flowering time control, and stress responses in plants.

## *In vitro* propagation and epigenetic variation

Plant tissue culture techniques have been developed and improved over time for numerous plant species. Many researchers have incorporated micropropagation techniques into many basic and applied aspects of plant science for years (Lee and Phillips, 1987). According to Cassells and Curry (2001), there are few well-described physiological, genetic, and epigenetic problems that are associated with a plant cultured *in vitro* such as recalcitrance, hyperhydricity, and somaclonal variation (Miguel and Marum, 2011). Variation that emerged in tissue culture plants has been termed “somaclonal variation” (Larkin and Scowcroft, 1981). Due to its origin, somaclonal variation is also referred to as tissue culture-induced variation (TCIV). Mutations are referred to as heritable genetic variations that emerged without any intervention by genetic recombination or segregation (Van Harten, 1998). Mutations can be induced either physically, chemically, or by tissue culture (Predieri, 2001; Roux, 2004). However, mutagenesis due to tissue culture is different because of physical and chemical agents, and is unclear in the long term (Larkin, 1998). *In vitro* propagation techniques such as callus induction, embryo formation, and regeneration can be exceptionally stressful to the plant cells (Lörz et al, 1988). TCIV can occur due to two reasons: epigenetic (developmental) and genetic (heritable variation) causes (Lörz et al, 1988). Epigenetic variations are more often transient and inheritable even though the plant material is asexually propagated (Duncan, 1996). However, these variations can last for many generations, and phenotypic variants involve changes in the expression of specific genes (Hartman and Kester, 1983). In multicellular organisms, genetic and epigenetic mechanisms are involved in coordinated gene expression. DNA methylation, chromatin modification, and non-coding RNA biosynthesis mediate epigenetic regulation (Bond and Finnegan, 2007).

**Figure 3:**
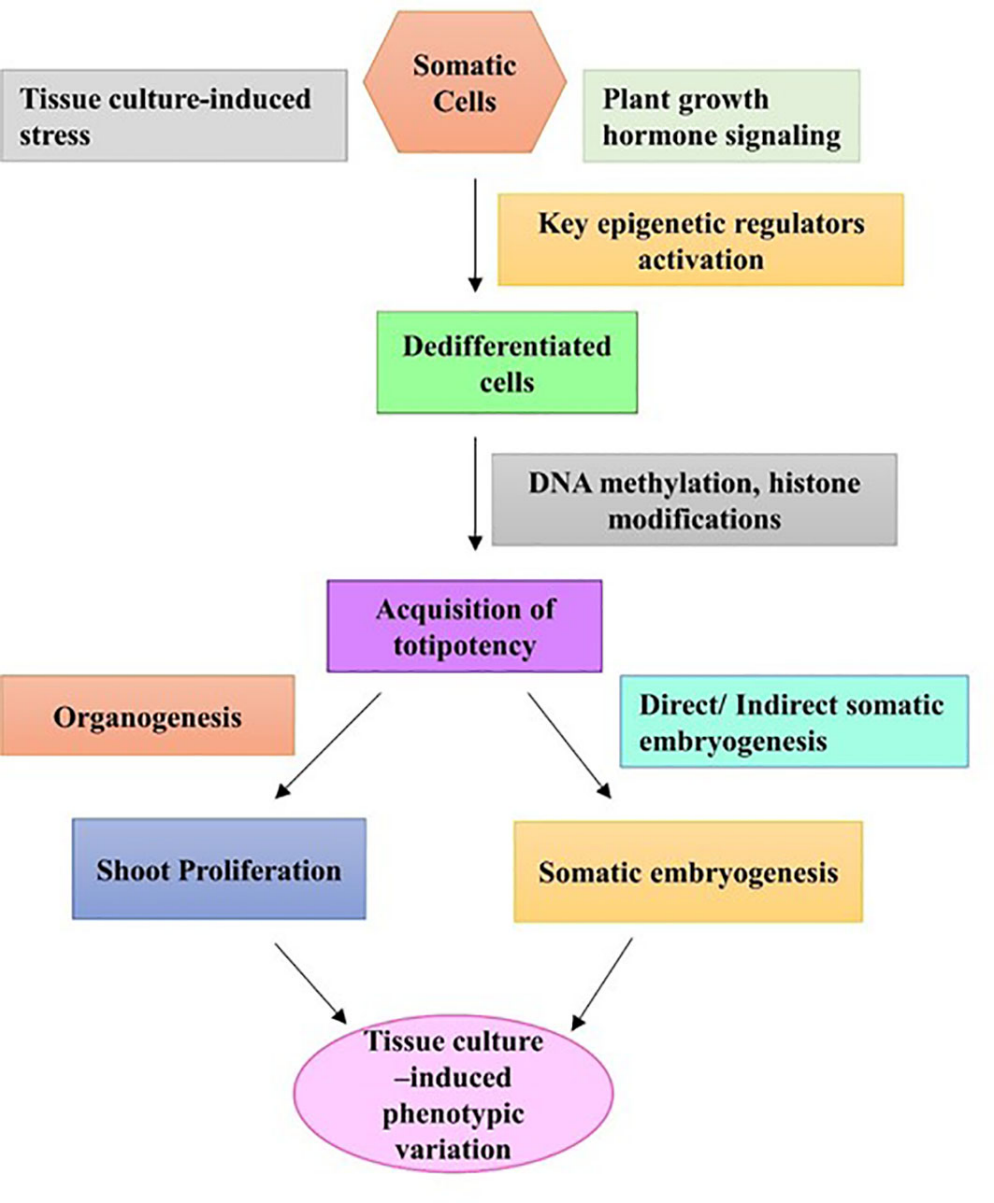
A hypothesized pathway of tissue culture-induced phenotypic variation due to activation of key epigenetic regulators.

Rejuvenation is described as the restoration of juvenile traits in an adult plant as a result of an epigenetic phenomenon. Many woody perennial plant species lose the ability to rejuvenate with increased maturity, which can impose a major problem in regard to vegetative propagation (Welander, 1983). However, according to Huang et al. (2012), a plant’s capability to rejuvenate can be gained during the tissue culture process. During the tissue culture process, plant cells go through callus formation (dedifferentiation) and plantlet regeneration (redifferentiation), which create a highly stressful condition for the plant material; thus, normal cellular regulation gets disrupted (Guo et al., 2007). It has already been determined that the degree of altered DNA methylation level is related to differentiation, and during dedifferentiation and redifferentiation, DNA methylation levels change drastically (Huang et al., 2012). There are a few studies available on DNA methylation during the tissue culture process. Goyali et al. (2015) observed that tissue culture-regenerated lowbush blueberry plants showed increased levels of antioxidant than their softwood cutting counterparts. Later, molecular analysis with simple sequence repeat (SSR) markers confirmed no genetic changes in the regenerants, which further led the authors to consider the involvement of epigenetic factors (Goyali et al., 2015). The first report of epigenetic variation through tissue culture-induced altered DNA methylation patterns was found in blueberry callus culture (Ghosh et al., 2017). A higher percentage of total DNA methylation was detected in callus tissues than in leaf tissues of three lowbush blueberry clones as well as in a hybrid genotype produced using the methylation-sensitive amplification polymorphism (MSAP) technique (Ghosh et al., 2017). It was also found that methylation events in *in vitro*-cultured calli were polymorphic compared to leaf samples from greenhouse-grown plants (Ghosh et al., 2017). TCIV arises due to changes in the microenvironment of the tissue culture system such as continuous subculturing and tissue culture-induced stress, and categorically includes molecular and physiological changes in the regenerants (**Figure 3**). Evidently, the occurrence of epigenetic variations such as DNA methylation is much more common in the tissue culture system and is thought to be one of the main causes of TCIV (reviewed by Ghosh et al., 2021b). Global methylation analysis using the MSAP technique detected altered DNA methylation patterns in tissue cultured plantlets of the lowbush blueberry clone “QB 9C” and the cultivar “Fundy” than in softwood cutting plants (Goyali et al., 2018). Shoots of two highbush blueberry cultivars, “Patriot” and “Chippewa”, derived from semisolid media and bioreactor systems, showed higher levels of methylation when compared with their greenhouse-grown donor plants. Among these two tissue culture systems, explants from the bioreaction system showed increased levels of methylation variation and polymorphism than explants derived from the semisolid media (Ghosh et al., 2021a). Cao et al. (2021) studied genomic DNA methylation using whole genome bisulfite sequencing (WGBS) in wild strawberry (*F. nilgerrensis*) at various tissue culture stages, starting from use of the shoot tip explants to out planting and acclimation. The authors found that the most obvious methylation changes occurred in the transposable element region of the genome, and the global methylation levels alternately increased and decreased during the tissue culture process. Additionally, during the dedifferentiation and redifferentiation stages of the tissue culture process, differentially methylated regions were detected, which were mostly transposable elements. This finding suggests that dedifferentiation and redifferentiation processes were involved in activation or silencing of transposable elements (Cao et al., 2021).

Sikdar et al. (2022) demonstrated the interaction between cytosine methylation and secondary metabolites in tissue-culture shoots, lingonberry plants, and cutting propagated plants under *in vitro* and *ex vitro* conditions. Through the MSAP assay, the authors observed more methylation in tissue culture shoots and plants than in the respective cutting propagated donor plants, although higher levels of secondary metabolites were observed in cutting propagated plants (Sikdar et al., 2022).

## Enhanced vegetative growth

Culture *in vitro* has significant influences on the growth and morphology of the resulting plants. Increased vegetative growth, branching, and rhizome production are often noted in tissue culture (TC) plants compared to conventional cutting propagated plants in berry crops. The variation in morphology between cutting propagated and tissue culture plants of berry crops has been reviewed elsewhere (Debnath et al., 2012; Debnath and Arigundam, 2020; Debnath and Goyali, 2020). Strawberry TC plants produced more crowns, leaves, runners, and berries than conventional runner cutting plants, which might be due to the plant growth regulator treatment used in culture media during *in vitro* culture (Debnath, 2009a; Debnath, 2017b). TC strawberries were more resistant to frost damage than the runner cutting plants (Rancillac and Nourrisseau, 1988). Likewise, Dalman and Malata (1997) observed that “Senga Sengana” TC strawberry plants were better for overwintering than those of runner propagated plants, although the opposite was true for the cultivar “Mari”, whereas for the cultivar “Jonsok”, there was no difference between the *in vitro* and *ex vitro* propagated plants. These results indicate that enhanced vegetative growth, berry production, and the development of TC plants over conventional cutting propagated plants, are genotype dependent. TC red raspberry plants produced longer and more numerous canes and more berries than those of root cutting propagated plants in the cultivar “Festival” but not in “Latham” (Debnath, 2014). Increased vegetative growth and rhizome production in TC plants were also reported in lingonberry (Debnath, 2005, 2006) and blueberry (Debnath, 2007). Cranberry TC plants had more runners, uprights, and leaves per upright than those of cutting propagated (CP) plants (Debnath, 2008). Micropropagated lowbush blueberry plants produced less flower buds than conventionally propagated plants (Jamieson and Nickerson, 2003).

Working with wild and cultivated lowbush blueberries over 5 years, Goyali et al. (2013) reported that TC plants produced a higher number of stems and branches with denser and larger shoots than the CP plants. Under field conditions, TC plants had longer canes with more berries and higher yields than the root cutting plants for the red raspberry cultivar “Festival” but not for “Latham” (Debnath, 2014). However, Naing et al. (2019) reported that meristem-derived micropropagated plants did not differ from those of conventionally propagated strawberry plants in terms of growth and fruit quality. The authors proposed that a low level of cytokinin (0.5 mg L^−1^ kinetin) in the culture medium might have produced phenotypically similar plants during micropropagation (Naing et al., 2019). Similarly, Arigundam et al. (2020) did not find any phenotypic difference between micropropagated and cutting propagated lingonberry plants. Cutting propagated lingonberry plants produced more vigorous growth but with less number of shoots, leaves, and rhizomes per plant compared to leaf and node culture-derived TC plants under greenhouse conditions (Sikdar et al, 2022). The juvenile characteristics of TC plants may be the reason for increased rhizome production, which is of great help for the quick establishment and early production of berry crops under field conditions (Debnath et al., 2012).

## Variation in bioactive compounds

Micropropagation of *Vaccinium* berry plants has been an area of interest for plant scientists due to possible implications for improving phytochemical properties. This technique has been used in several medicinal plant species to elevate the levels of phenolic components with antioxidant activities (AAs) to satisfy industrial pharmaceutical needs (Giri and Zaheer, 2016). It was found earlier that micropropagated berries have a higher level of antioxidants in comparison to conventionally propagated blueberries (Debnath and Goyali, 2020). However, micropropagated lingonberry plants had less chlorophyll a and b contents in the leaves compared to cutting propagated plants (Arigundam et al., 2020). The reason behind the different levels of bioactive compounds during plant tissue culture can be due to the use of PGR in the growth media, which may be involved in the up- and downregulation of genes engaged in secondary metabolite production pathways (Zifkin et al., 2012).

Blueberries are popularly known as a “super fruit” because of their high levels of *in vitro* antioxidant properties due to the ample presence of polyphenolic compounds (Kalt et al., 2020). However, the antioxidant capacities of these phenolic compounds were not directly accessible under *in situ* conditions due to their low bioavailability (Williamson and Clifford, 2010). In blueberries, the antioxidant capacity depends on the presence of polyphenolic and flavonoid compounds, their redox potential, and structures (Prior et al., 1998). It was previously proven that there was a strong and positive correlation between the total phenolic (TPC) and anthocyanin content (TAC) and AA in blueberries (Goyali et al., 2013, 2015). However, the relationship between TPC and AA was much more stronger than TAC and AA (Moyer et al., 2002). Moreover, Ghosh et al. (2018) reported that in half-high blueberry plants, TPC and AA were strongly related in comparison to the total flavonoid content (TFC) and AA. Nonetheless, quantification of total AA in a plant is a complex process as it is influenced by the linkage of different phytochemicals and works synergistically or antagonistically in the presence of various environmental factors (Hassimotto et al., 2005).

It was proven that the type of cytokinin and concentration in the culture media affects the biosynthesis pathways of secondary metabolites (Debnath and Goyali, 2020). Phenolic compounds are the most commonly found secondary metabolites in plants, and are derivatives of phenylalanine. Cytokinin concentration is positively correlated with the level of transcription of gene-encoding enzymes such as ammonia lyase, chalcone synthase, chalcone isomerase, and dihydroflavonol reductase involved in the flavonoid biosynthesis pathway, and is thus indirectly correlated to the TAC in Arabidopsis (Deikman and Hammer, 1995). In addition, various environmental factors like low concentrations of nutrients and light increase phenylalanine ammonia lyase activity, which is an important regulatory factor of plant metabolic pathways (Taiz and Zeiger, 2006). It was found that the lowbush blueberry clone “QB9C” and the cultivar “Fundy” were affected by micropropagation techniques and “QB9C” fruits showed a higher content of secondary metabolites and AA than “Fundy” regenerants (Goyali et al., 2013). Later, Goyali et al. (2015) reported increased levels of TPC, TAC, TFC, total proanthocyanidins (TPAC), and AA in the tissue culture regenerants of the lowbush blueberry clone “QB9C” and the cultivar “Fundy”, in comparison to stem cutting counterparts. Georgieva et al. (2016) studied the TPC, AA, and ferric-reducing capacities of *in vitro*- and *ex vitro*-grown lingonberry, bilberry, raspberry, and strawberry fruit extracts. The authors found a high content of AAs in all three types of *in vitro*-grown berries in comparison to berries collected from the *ex vitro* condition. Bioreactor- and agar-gelled media-derived strawberries of the cultivar “Bounty” exhibited a higher content of TAC and AA than strawberries grown using conventional runner cuttings. Even though there was a significant difference in the AAs of the fruits collected from two different pathways of tissue culture and conventional cuttings, the inter-simple sequence repeat (ISSR) marker assay did not show any heterogeneities in their amplification profiles. These results confirmed the clonal fidelity of the micropropagated plants, yet suggest the occurrence of somaclonal variation as a reason for increased TAC and AA levels (Debnath, 2009a). Similar results were also reported in lingonberry wild clones, where the clonal fidelities of TC plants were confirmed by expressed sequence tag (EST)–SSR, EST–polymerase chain reaction (PCR), and ISSR markers; however, variation was observed between the chlorophyll contents of TC and CP plants (Arigundam et al., 2020).

## Detection of DNA methylation in tissue culture-derived berry plants

There are various technologies available to evaluate the role of epigenetic changes in a crop improvement program such as the use of modern genome editing tools like CRISPR/Cas9, zinc finger nucleases, transcription activator like-nucleases, epigenetic recombinant inbred lines, and RNA-directed DNA methylation (Arencibia et al., 2019). However, the study of global DNA methylation can be employed in a small-scale tissue culture set up to understand the alteration of the epigenetic status of regenerants. DNA methylation studies can be approached from various standpoints as there are different methods available to detect tissue culture-induced DNA methylation and localization of methylated cytosine in a particular region of the genome. Among them, the most popular are detection by MSAP, high-performance liquid chromatography (HPLC), high-performance capillary electrophoresis (HPCE), and WGBS. MSAP is based on the sensitivity of two restriction enzymes (*Msp*I and *Hpa*II) to identify methylated cytosine bases (Cedar et al., 1979). This is a modification of the amplified fragment length polymorphism (AFLP) technique (Vos et al., 1995), which was developed by Reyna-Lopez et al (1997) to detect DNA methylation in dimorphic fungi. The MSAP technique has been used in tissue culture regenerants to detect global methylation in only a few berry crops including blueberry (Ghosh et al., 2017; Goyali et al., 2018; Ghosh et al, 2021a), grapes (Baránek et al., 2010), and lingonberry (Sikdar et al., 2022). The genomic DNA level of methylated cytosine can be detected by enzymatic means, although this is not as sensitive as the HPLC method because its resolution is restricted to the cleavage sites of the endonucleases. The HPLC technique quantifies DNA methylation *via* fractionation of four main bases (A, T, G, and C) (Fraga and Esteller, 2002). When options are available, HPCE proves to be more beneficial than HPLC as it is faster, cheaper, and comparatively more sensitive (Fraga et al., 2000). These methods have not been used in berry crops to date, but have been used to detect cytosine methylation in tissue culture regenerants in various other crops such as oil palm (Jaligot et al., 2000), *Acca sellowiana* (Fraga et al., 2012), triticale (Machczyńska et al., 2014), and apple (Li et al, 2002). Because of these benefits, enzymatic methods are commonly used, as they do not involve complex equipment or skilled labor. Enzymatic isoschizomer-based methods do not provide any details on the role of methyl cytosine in cell and molecular biology. However, these details can be determined with the use of bisulfite modification of methylated DNA. There are various methods available based on the treatment of sodium bisulfite to detect methylated cytosine located at specific locations in DNA (Fraga et al., 2000). Sodium bisulfite converts unmethylated cytosine to uracil, whereas methylated cytosine stays the same (Furiuchi et al., 1970). WGBS provides genome-wide methylation profiling without any interference; however, not much information is available on the minimum required coverage and other factors such as susceptibility, precision, and cost of the assay (Ziller et al., 2015). WGBS has been used in wild strawberry (Cao et al., 2021) and in blueberry tissue culture (Ghosh et al., unpublished results).

## Conclusions

Micropropagation techniques for the improvement and commercial production of berry plants are currently used as alternative methods to satisfy market demand. In plants, gene regulation is related to the level of DNA methylation, and this epigenetic mechanism is intricately linked to growth and development, as well as *in vitro* processes such as organogenesis and SE. It is important to further explore the epigenomes of berry plants to see if an epigenetic footprint remains within the epigenome of regenerated plants due to different culture environments. This can be done by identifying genes subjected to differential methylation, identifying regions of differential methylation, and by identifying the hypo/hypermethylated gene sets involved in various biological and molecular functions under *in vitro* and *in vivo* conditions using advance methylation detection techniques. In a crop improvement program, it is important to employ genome editing tools such as CRISPR/Cas9 to detect the occurrence of undesirable traits due to epigenetic variation during the tissue culture process as well as to apply various bioinformatic tools to predict the inheritance of altered epigenetic patterns in regenerants. Enhanced vegetative growth and/or bioactive components due to *in vitro* culture and propagation are of significant importance for early field establishment and improved berry production, and for increased health-promoting factors in berry crop production and improvement programs.

## Author contributions

Conceptualization, writing, reviewing and editing by SD. Writing and reviewing by AG. Both authors agree with the final version of the manuscript. The authors declare that the content of this paper has not been published or submitted for publication elsewhere. All authors contributed to the article and approved the submitted version.
